# Bioremediation of aquaculture wastewater using the fungal biomass integrating Plackett–Burman design

**DOI:** 10.1007/s10532-025-10222-5

**Published:** 2025-12-05

**Authors:** Hazem T. Abd El-Hamid, Muhammad A. El-Alfy, Hanan M. Hafiz, Hoda M. El-Gharabawy

**Affiliations:** 1 National Institute of Oceanography and Fisheries, NIOF, Cairo, Egypt; 2 Botany and Microbiology Department, Faculty of Science, Damietta University, New Damietta, 34517 Egypt

**Keywords:** Mycoremediation, Aquaculture effluent, Plackett–Burman design, Nutrient removal, *Aspergillus niger*, Phosphorus reduction, Fungal Biotreatment

## Abstract

Aquaculture wastewater contains elevated levels of nutrients and organic pollutants that can accelerate eutrophication and impair aquatic ecosystems if discharged untreated. In the study, a fungal-based remediation approach was investigated for the removal of pollutants from aquaculture wastewater collected from Baltim Station ponds (31.55244° N, 31.092855° E) near Lake Burullus, Egypt. Two native fungal isolates, *Aspergillus niger* and *Aspergillus flavus*, were employed for primary mycoremediation experiments, while *Ganoderma mbrekobenum* was included only in the Plackett–Burman experimental design to evaluate the influence of environmental and nutritional factors on total phosphorus (TP) removal under optimized conditions. The fungal treatment significantly improved water quality, showing substantial reductions in total protein, phosphorus, nitrogen, organic matter, and chemical oxygen demand (COD) indicating a vital role of *Aspergillus* species in the bioremediation of nutrient-rich aquatic environments. The Plackett–Burman design (PBD) showed that fungal treatment significantly reduced pollutant concentrations with higher metabolic activity and enzymatic production as dehydrogenase and total protein from 9 to 12 days. Moreover, PBD identified KH_2_PO_4_ and MgSO₄ as the most influential variables for enhancing TP removal in the presence of *G. mbrekobenum*, while peptone and yeast extract exhibited the greatest effect in the non-fungal control system. The regression models demonstrated strong predictive accuracy (R^2^ > 0.99), confirming the validity of the optimization approach. The results highlight the effectiveness of fungal biomass as a cost-effective and eco-friendly bioremediation strategy for mitigating nutrient pollution in aquaculture effluents and protecting sensitive aquatic environments such as Lake Burullus.

## Introduction

Aquaculture wastewater is increasingly recognized as a major contributor to nutrient loading in freshwater and coastal environments, often exerting ecological pressures comparable to those of municipal and industrial effluents (Yoboue et al., 2018; Ghyadh et al. 2019). When discharged without adequate treatment, these effluents can negatively impact surface water quality, alter the physicochemical balance of receiving water bodies and accelerate eutrophication processes, resulting in harmful algal blooms, oxygen depletion, habitat degradation, and loss of biodiversity (Korboulewsky et al. 2007; El-Alfy et al. 2021, 2025). The environmental vulnerability of Lake Burullus; a shallow coastal lagoon situated between the Rosetta and Damietta branches of the Nile Delta—makes it particularly sensitive to nutrient-enriched aquaculture discharges. The lake lies between longitudes 30°30′–31°10′E and latitudes 31°19′–31°36′N and is of substantial ecological and socio-economic importance.

Conventional physicochemical treatment methods are costly require intensive infrastructure, and may generate secondary pollutants. In contrast, biological remediation offers a more sustainable solution due to their efficiency, low operational cost, and minimal ecological footprint. Fungal bioremediation (Myco-remediation) has attracted growing interest because filamentous fungi possess highly adaptable metabolism and a broad enzymatic repertoire capable of degrading or transforming complex organic compounds and assimilating inorganic nutrients (Zeyaullah et al. 2009; Kuppan et al. 2024; Gharieb et al. 2024). Kshirsagar (2013) used aquatic fungi, specifically *Aspergillus terreus*, *Aspergillus niger*, *Rhizopus nigricans*, and *Cunninghamella* sp., in a bioremediation process for wastewater treatment. In particular, species of *Aspergillus* and *Ganoderma* produce extracellular hydrolytic and oxidative enzymes that facilitate the breakdown, assimilation, and mineralization of nitrogenous and phosphatic compounds, as well as high-molecular-weight organic matter (Bhandari et al. 2021; Li and Wang 2023).

Despite the demonstrated potential of fungal systems in wastewater treatment, applications in aquaculture effluent remediation remain comparatively underexplored. Limited research has focused on the use of fungal biomass, particularly native fungal strains, as an efficient and eco-friendly bioremediation tool. Most prior work has focused on bacterial and algal bioreactors, while the biotechnological capacities of filamentous fungi have not been sufficiently optimized (Mohsenpour et al. 2021). Moreover, the efficiency of fungal bioremediation is strongly influenced by environmental and nutritional conditions; therefore, rational optimization frameworks are essential to define the most influential variables governing pollutant removal. The Plackett–Burman screening design provides an effective statistical approach for identifying significant operational factors among multiple candidates, thereby minimizing experimental effort and guiding subsequent optimization strategies (Abd El-Hamid et al. 2018; Vanaja and Rani 2008).

The present study investigates the mycoremediation potential of native *Aspergillus niger* and *Aspergillus flavus* isolated from aquaculture wastewater and evaluates their capacity to remove total phosphorus (TP), total nitrogen (TN), chemical oxygen demand (COD), and oxidizable organic matter (OOM). In addition, a previously characterized strain of *Ganoderma mbrekobenum*—a wood-decaying Basidiomycete—was included exclusively in the Plackett–Burman statistical optimization experiment to determine the key environmental and nutritional variables influencing TP removal efficiency. This approach establishes a methodological integration between empirical bioremediation trials and data-driven optimization.

This research aims to (i) assess the pollutant removal efficiency of native *Aspergillus* species in aquaculture wastewater, (ii) determine the metabolic and enzymatic responses associated with organic and inorganic nutrient reduction, and (iii) identify the critical factors influencing phosphorus removal under fungal mediation. By linking fungal bioremediation performance to optimized operational conditions, this study contributes to the development of cost-effective, scalable, and environmentally sustainable treatment strategies for aquaculture effluents, with direct relevance to the conservation of sensitive coastal ecosystems such as Lake Burullus.

## Materials and methods

### Aquaculture wastewater collection

Aquaculture wastewater was collected in August 2024 from fish ponds at the Baltim station located near the southeastern part to the Lake Burullus (31.55244° N, 31.092855° E). The sample container was rinsed several times with the wastewater before sampling to avoid cross-contamination. About 10 L of wastewater were collected and transported to the laboratory under cooled conditions. The initial physicochemical characteristics of the wastewater were recorded as follows: temperature 26 ± 2 °C, pH 7.5 ± 0.1 and salinity 32.0 ± 1.0 ppt.

### Fungal isolation and preservation

Fungal isolation was performed using DOX agar medium (20 g Sucrose, NaNO_3_ 3 g, KCl 1 g, MgSO_4_.7H_2_O 0.5 g, KH_2_PO_4_.5H_2_O 0.5 g, 15 g Agar, 1L dist. H_2_O). Dox agar medium was prepared in a 500 mL conical flask. All glassware and media were sterilized by autoclaving at 121 °C and 1.5 bar (approximately 15 psi) for 20 min, then supplemented with trace streptomycin antibiotic, and poured into sterile Petri plates (9 cm) and left to solidify under aseptic conditions. 1 mL of wastewater samples was used as inoculum for each plate by spread plate method, then plates were incubated at 30 ºC for 3–5 days to observe any fungal growth. The growing fungal colonies were then separated into pure cultures and preserved on DOX slopes at 5 ºC and in 20% Glycerol vials at -80 ºC for subsequent analyses.

### Morphological identification of the fungi

The identification of isolated fungi (F1 and F2) was carried out based on both macro and micro-morphological characterization of the pure cultures on DOX plates (7 days old) following standard mycological keys (Samson and Varga 2009; Smith 2012; Afzal et al. 2013). The macro-morphology included colony texture, pigmentation, and margin characteristics, presence and absence of exudates, etc. Microscopic structures were examined on slides under a light microscope using lactophenol-cotton blue dye, and characters as mycelial branching, head shape, conidiophore shape, spore arrangement, wall texture and other relevant characteristics were recorded at 400 and 1000 X magnification power.

### Myco-remediation test

The collected wastewater samples were used to prepare two treatment groups: (i) without pH adjustment and (ii) with pH adjusted to 6.5 ± 0.2. Each treatment was further subdivided into three experimental sets to conduct parallel physicochemical and biological analyses. For fungal inoculation, two mycelial discs (5 mm diameter) from actively growing 5-day-old cultures of each fungal isolate (F1 and F2) were transferred separately into 1000 mL Erlenmeyer flasks containing 250 mL of wastewater. A third set of flasks containing wastewater without fungal inoculation served as the control. Flasks were incubated at 28 ± 2°C for 12 days. Samples were drawn at 3-day intervals for physicochemical and enzymatic analyses.

### *Ganoderma mbrekobenum* for optimization experiments

**Fig. 1:**
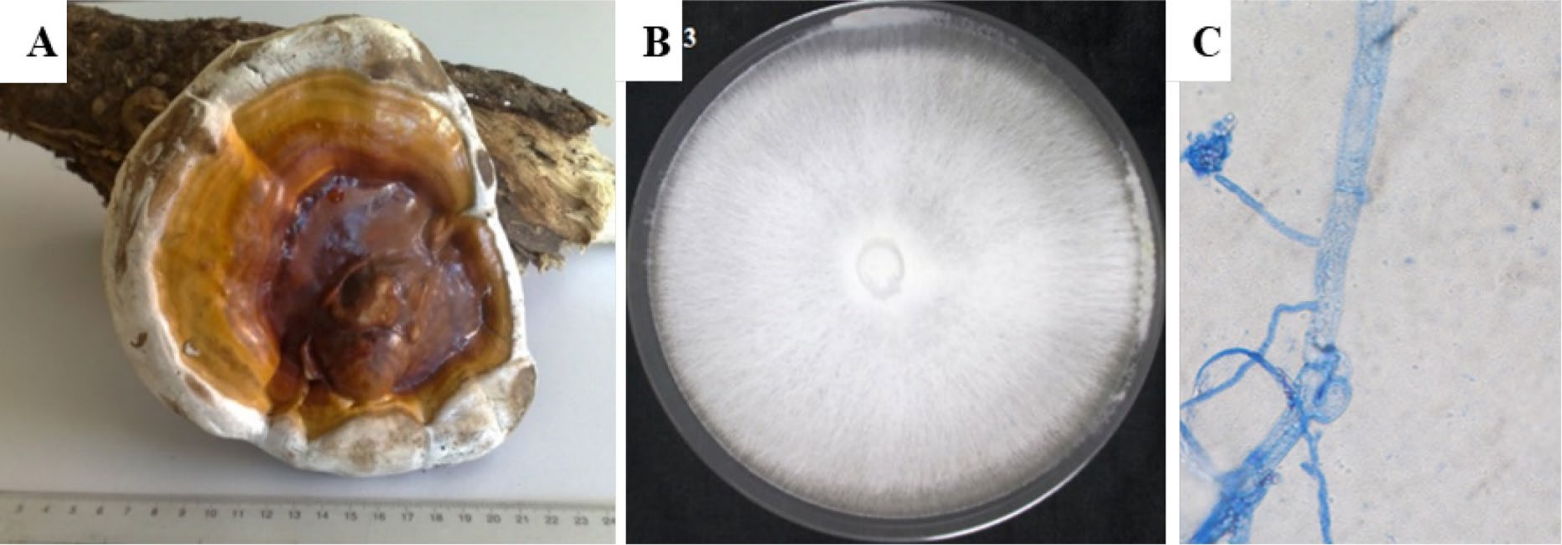
*Ganoderma mbrekobenum* EG-HE01 used in the study; **A** Fruiting body naturally occurring on a decayed *Citrus limon* trunk (Damietta, Egypt). **B** Pure white mycelial culture grown on PDA (5 days old). **C** Microscopic hyphae showing clamp connections characteristic of Basidiomycota. Strain identity confirmed by DNA-ITS sequencing (GenBank Accession: PX240414)

A previously isolated and molecularly characterized strain of *Ganoderma mbrekobenum* (El-Gharabawy 2016) (GenBank Accession: PX240414), originating from decayed *Citrus limon* wood in Damietta, Egypt, was used for this experiment. It was included solely in the Plackett–Burman optimization experiment to assess the influence of environmental and nutritional parameters on fungal-mediated phosphorus removal. The strain was cultured on potato dextrose agar (PDA; Potato extract 200 g, dextrose 20 g, agar 15 g, 1 L dist. H_2_O) at 25 °C for 5 days prior to use until complete colony growth (**see **Fig. 1). Each experimental flask contained 250 mL of wastewater sample with  two discs (5 mm diameter) inoculum of actively growing fungal biomass. All experiments were performed in triplicate to ensure reproducibility.

### Physicochemical analyses

Total phosphorus (TP) and total nitrogen (TN) were analyzed following Grasshoff et al. (1999). Oxidizable organic matter (OOM) was measured using the permanganate oxidation method (FAO 1975). Chemical oxygen demand (COD) was determined using the dichromate reflux method (APHA 1998). The pollutant removal efficiency during fungal incubation periods was calculated by the following equation:

$${\mathrm{Re}} moval effeciency \% = (C_{i} - C_{f} )/C_{i} x100$$

where: C_i_ = initial concentration of the pollutant (beginning of period) and C_f_ = final concentration of the pollutant (end of period).

### Enzymatic activity

#### Dehydrogenase and protein measurements

Dehydrogenase activity was measured using the triphenyl tetrazolium chloride (TTC) reduction assay (Alef 1995). Samples were incubated for 24 h at 37 °C and absorbance measured at 485 nm. Total protein content was measured using Bradford assay (Bradford 1976) by adding 1 mL of sample to 3 mL of protein reagent and absorbance was measured at 595 nm.

### Plackett–Burman screening design

The Plackett–Burman Design was adopted to statistically screen key operational parameters influencing the efficiency of fungal bioremediation. Although wastewater characteristics vary, the chosen variables; pH, temperature, inoculum size, agitation speed, carbon source, nitrogen source, and incubation time represent major environmental and nutritional factors directly affecting fungal growth and pollutant removal capability. This design enables the identification of the most significant factors among multiple variables, reducing experimental trials and providing a foundation for subsequent optimization (Hegazy et al. 2015). The Plackett–Burman design (PBD), developed by Plackett and Burman (1946), is a widely used class of screening designs. It focuses on improving quality control processes by studying the effects of various parameters (Vanaja and Rani 2008).

The Plackett–Burman experimental design is based on the following first-order model:

$$Y \, = \, \beta 0 \, + \, \Sigma \, \left( {\beta iXi} \right)$$

where: Y is the response (e.g., productivity or specific activity), β0 is the model intercept, βi is the linear coefficient for the i-th variable, and Xi is the level of the i-th independent variable.

This model helps identify the key variables that contribute most significantly to maximizing productivity. In this study, ten variables (medium constituents or environmental factors) were screened using twelve experiments (Table 1). Variables exhibiting high confidence levels are considered significant and have a substantial impact on the bioremediation process (Mabrouk et al. 2012; Abd El-Hamid 2015).

**Table 1:** Medium and variables used in the Plakett-Burman design

Run	TºC	pH	Glucose	Peptone	Yeast	NaNO_3_	NH_4_Cl	(NH_4_)_2_SO_4_	KH_2_PO_4_	MgSO_4_·7H_2_O
1	–1	1	1	1	–1	–1	–1	1	–1	1
2	1	–1	1	1	–1	1	1	1	–1	–1
3	–1	1	–1	1	1	–1	1	1	1	–1
4	–1	–1	–1	–1	–1	–1	–1	–1	–1	–1
5	1	–1	–1	–1	1	–1	1	1	–1	1
6	1	–1	1	1	1	–1	–1	–1	1	–1
7	–1	–1	–1	1	–1	1	1	–1	1	1
8	–1	1	1	–1	1	1	1	–1	–1	–1
9	1	1	–1	1	1	1	–1	–1	–1	1
10	–1	–1	1	–1	1	1	–1	1	1	1
11	1	1	–1	–1	–1	1	–1	1	1	–1
12	1	1	1	–1	–1	–1	1	–1	1	1

### Statistical analysis

One-way ANOVA and Dunn’s post hoc analyses were used to compare several and individual means, respectively. It was conducted using the PAST program. The negative values of TP removal percent were excluded in Blacket Burman experiment, so it was excluded from the ANOVA analysis to match the experimental work design.

## Results and discussion

### Fungal identification

**Fig. 2:**
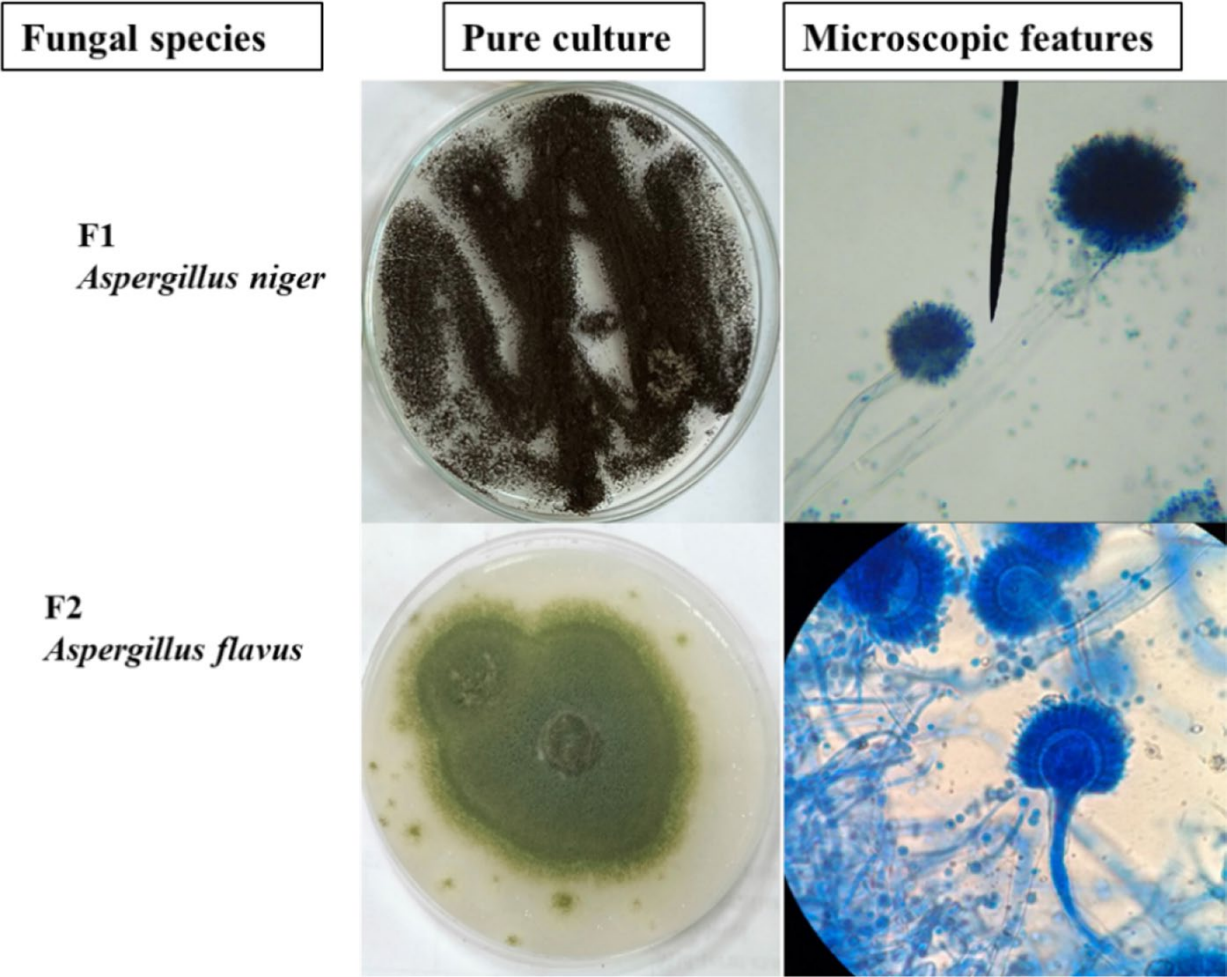
Morphological characters of the pure colony of two isolated fungal species from water sample; F1 = *Aspergillus niger*, F2 = *Aspergillus flavus*

The isolation and morphological identification revealed two distinct fungal species of the Ascomycete group: *Aspergillus niger* (F1) and *Aspergillus flavus* (F2) (Fig. 2) were obtained from the aquaculture wastewater samples. The first isolate was fast-growing, dark brown to black colonies with a white thin margin, and no exudates nor reverse was observed. Under microscope; conidiophore was smooth walled; conidial heads were globose and radiate, vesicles were circular and up to 80 µm, conidia were brown, globose and up to 4.2 µm in diameter. The second isolate displayed a yellow olive to green colony, with a thin white margin and moderate growth rate, and no exudates nor reverse was observed. Under microscope; conidiophores were long and colorless, head was radiate with biseriate phialide and small, vesicle sub-globose, and conidia were globose and partially roughened.

The presence of these species is consistent with previous reports documenting *Aspergillus* spp. as dominant fungal taxa in aquaculture wastewater and organically enriched aquatic environments due to their capacity to utilize dissolved nutrients and suspended organic matter as carbon sources (Kuppan et al. 2024).

### Mycoremediation of aquaculture wastewater

The experimental results revealed that both *A. niger* and *A. flavus* significantly improved water quality relative to the untreated control (Table 2), demonstrating the capability of fungal biomass to reduce organic and inorganic loads in aquaculture wastewater (Akpasi et al. 2023). Marked reductions were observed in total phosphorus (TP), total nitrogen (TN), chemical oxygen demand (COD), and oxidizable organic matter (OOM) over the 12-day incubation period.

**Table 2:** Pollutant removal efficiency (%) for used fungal Species

Parameter	Treatment	Optimal removal Period (Days)	Optimal removal day	Removal (%)
Total protein	*A. flavus (* +*)*	3–9 days	9	66.30%
	*A. niger*	3–9 days	9	66.50%
	*A. niger (* +*)*	3–9 days	9	63.70%
Dehydrogenase	*A. flavus (* +*)*	3–6 days	6	9.60%
	*A. niger*	3–6 days	6	2.20%
	*A. niger (* +*)*	3–3 days (no change)	3	~ 0%
Total phosphorus (TP)	*A. flavus (* +*)*	3–6 days	6	60.40%
	*A. niger*	3–6 days	6	93.60%
	*A. niger (* +*)*	3–12 days	12	17.20%
Total nitrogen (TN)	*A. flavus (* +*)*	3–6 days	6	54.20%
	*A. niger*	3–6 days	6	56.00%
	*A. niger (* +*)*	3–6 days	6	55.30%
Chemical oxygen demand (COD)	*A. flavus (* +*)*	3–6 days	6	35.90%
	*A. niger*	3–12 days	12	22.20%
	*A. niger (* +*)*	3–12 days	12	32.30%
Organic matter (OM)	*A. flavus (* +*)*	3–3 days (no change)	3	0%
	*A. niger*	3–6 days	6	16.70%
	*A. niger (* +*)*	3–12 days	12	100%

**Fig. 3:**
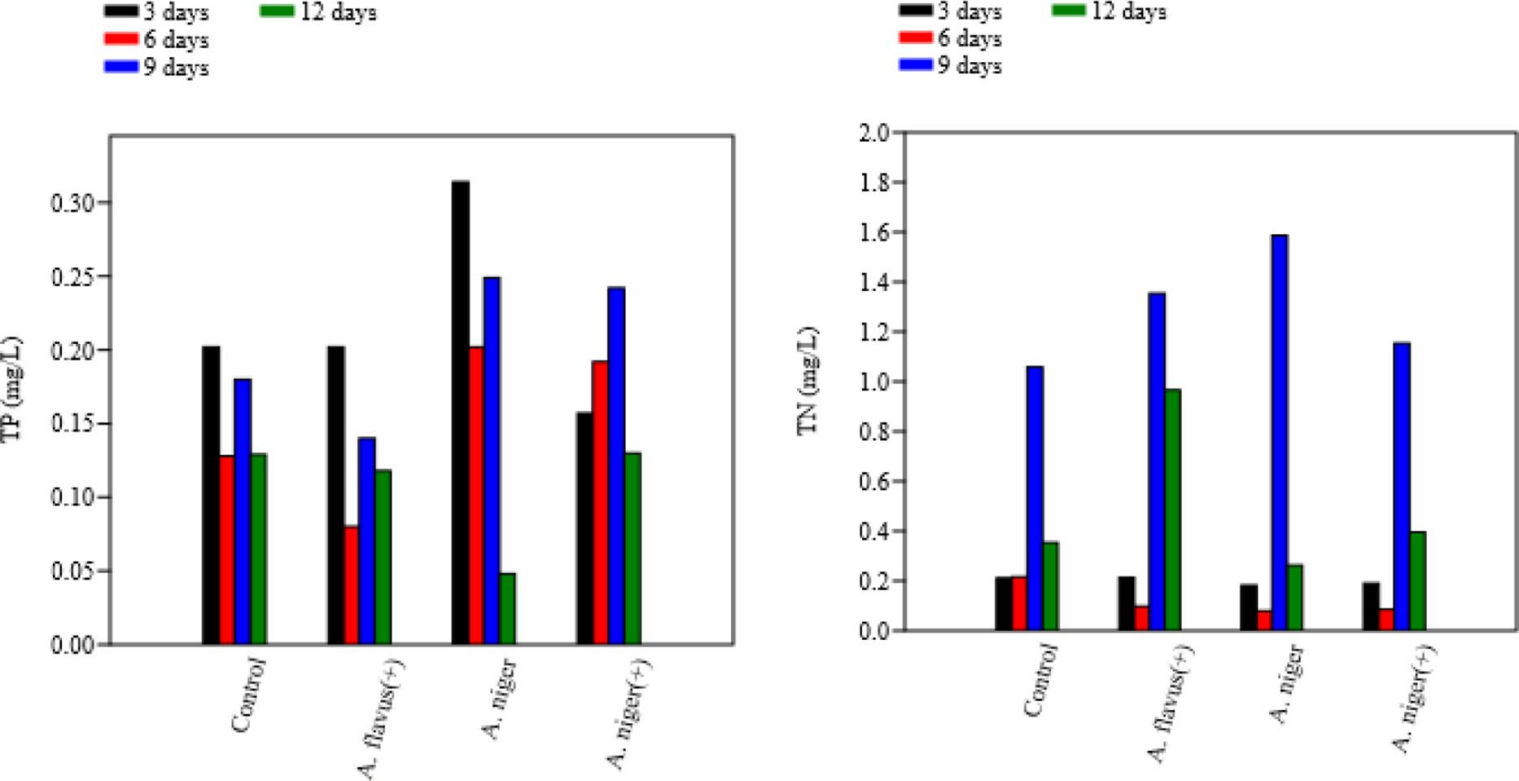
Measured concentrations of TP and TN through remediation using *A. flavus* and *A. niger* with ( +) and without pH adjustment

The most notable change was observed in TP which showed a steady decline beginning at day 3 and reaching maximum removal between days 9 and 12 (Fig. 3). This removal trend aligns with the fungal growth phase, during which phosphorus is assimilated to support biomass development and energy metabolism (Dalecka et al. 2020). COD and OOM reductions also followed similar temporal profiles, reflecting progressive enzymatic oxidation of dissolved and particulate organic matter.

**Fig. 4:**
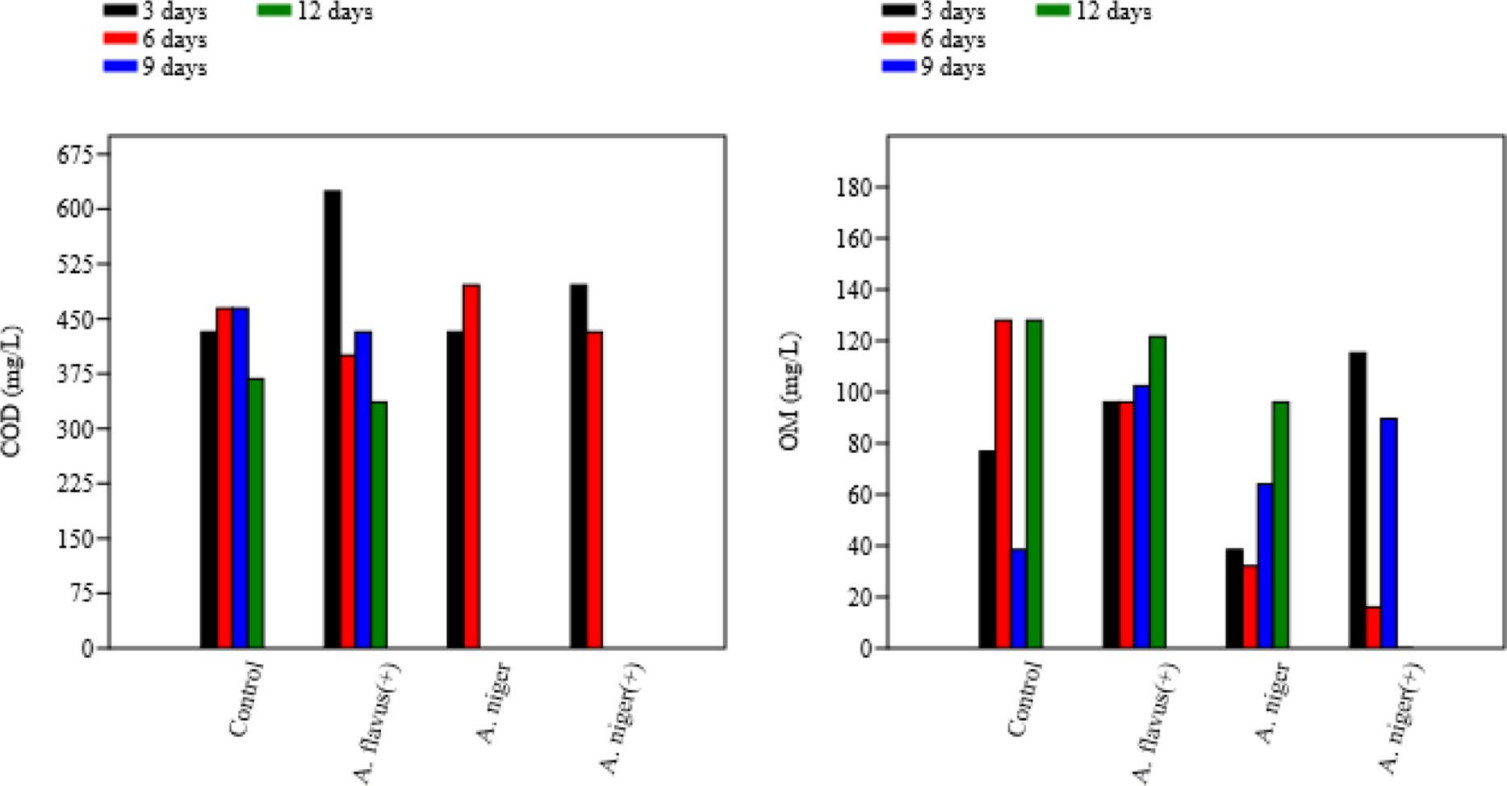
Measured concentrations of COD and OM through remediation using *A. flavus* and *A. niger* with ( +) and without pH adjustment

Interestingly, *A. flavus*, however, showed a different trend, with OM concentration increasing over time, and the best OM removal observed at 3 days of incubation as shown in Fig. 4. It’s obvious that in case of *A. flavus* it showed high removal of COD (low concentrations within time) met high concentration of OM in samples within time. It could be interpreted by that the fungal enzymatic system converts the complex and harmful industrial pollutants into harmless by-products measured as OM in samples, thus reducing the impact on the environment and ecosystem (Latif et al. 2023).

When *A. niger* was tested without pH adjustment, the most effective removal of TP and COD occurred after 12 days, whereas, the lowest TN and OM concentrations were observed after 6 days. Previous studies support these finding; *A. niger* can significantly reduce phosphate concentrations in wastewater between 48—96 h at temperatures of 30–45 °C (Akpor et al. 2013) and its growth is favoured at 30–37 °C (Mogensen et al. 2009).

Although both species improved wastewater quality, *A. niger* consistently demonstrated higher removal efficiency, particularly for TP and OOM. The superior performance of *A. niger* may be attributed to: (i) higher extracellular phosphatase production, enabling more rapid hydrolysis of inorganic phosphate complexes, (ii) greater ability to form dense mycelial networks, enhancing biosorption of dissolved nutrients or (ii) higher metabolic rate under mildly acidic conditions, consistent with the adjusted pH treatment. Similar findings describe *A. niger* as a robust strain for nutrient-rich wastewater remediation (Gharieb et al. 2024).

Other studies further support fungal wastewater treatment efficiency. Kadhim et al. (2021) demonstrated the effectiveness of *A. niger*, *A. terrues*, and *P. digitatum* in wastewater remediation, achieving reductions of 87–97% for NO_3_ and 22.8–32.1% for PO_4_. The relatively low phosphate removal can be attributed to the limited phosphate assimilation capacity of the fungal strain under the tested conditions. Unlike nitrogen and organic compounds, phosphorus removal largely depends on chemical precipitation or intracellular storage mechanisms, which were not predominant in this treatment system. Moreover, the available carbon–phosphorus ratio may not have favored optimal phosphate uptake by fungal biomass. *A. flavus* has also shown strong BOD reduction in dairy effluents (Chaithra et al. 2023). *A. niger* reduced COD in olive mill wastewater by 80% after an adaptation period (Aissam et al. 2007), while Coulibaly et al. (2003) reported COD and TN removal rates of 72% and 65.4%, respectively.

White-rot and filamentous fungi produce extracellular enzymes such as ligninases, cellulases, amylases, oxygenases, and dehydrogenases that degrade complex organic pollutants (Zhang et al. 2024; Baffi et al. 2024). Dehydrogenases, in particular, are key in degrading aromatic pollutants and dyes (Bhandari et al. 2021). Ramzan et al. (2025) further reported strong negative correlations between COD/nutrient levels (PO_4_, NO_2_, NO_3_ and NH_4_) and fungal metabolic activity in aquaculture wastewater.

### Enzymatic and metabolic response during bioremediation

**Fig. 5:**
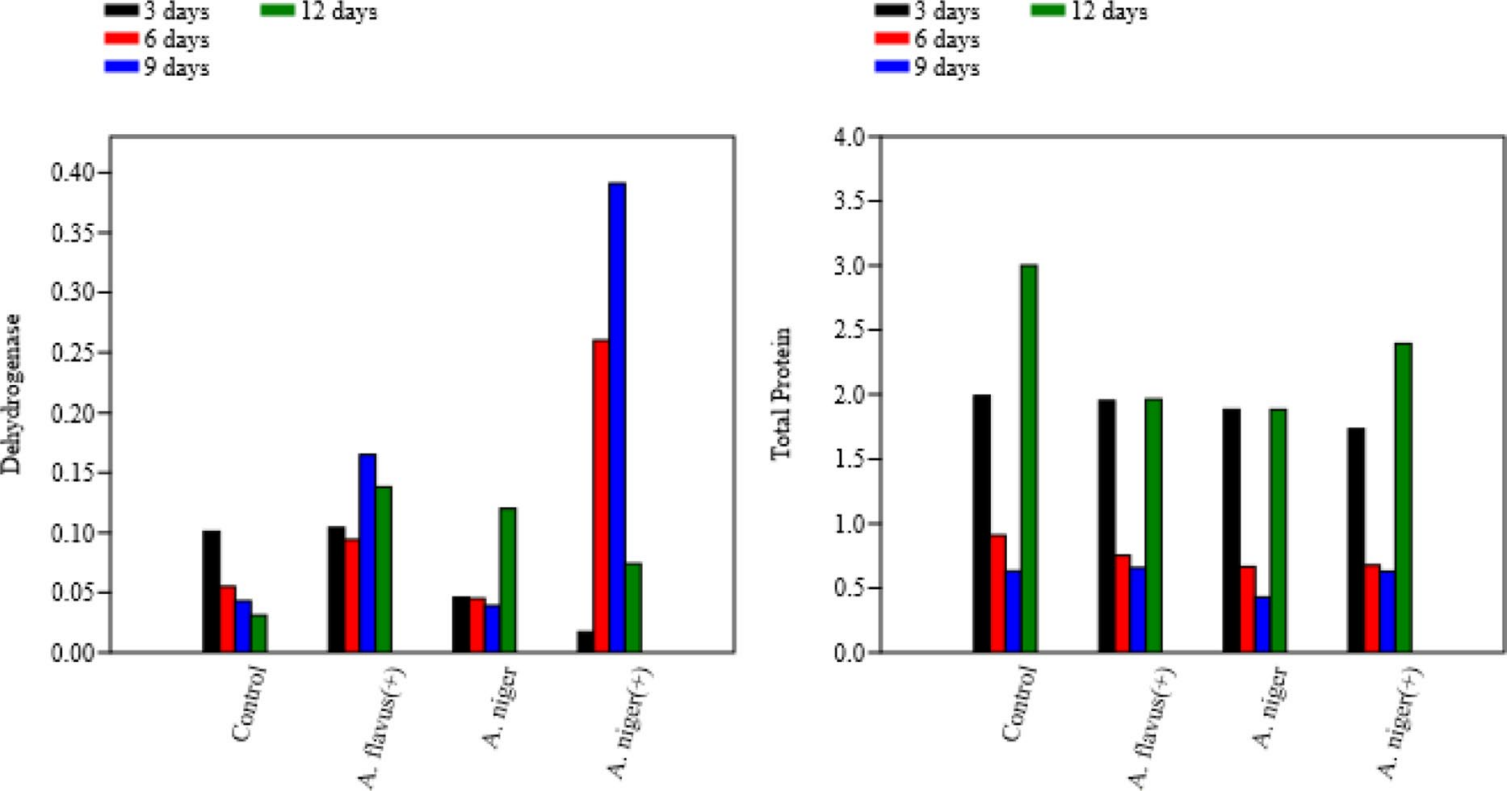
The metabolic rate for enzymes with ( +) and without pH adjustment through incubation time

The metabolic activity of the fungal biomass was evaluated through total protein content and dehydrogenase enzyme activity under both pH-adjusted and non-adjusted conditions. The highest total protein levels, indicative of maximum fungal growth and metabolic activity, were recorded after 12 days of incubation for all fungal treatments. For dehydrogenase activity, the highest enzymatic turnover in pH-adjusted treatments occurred after 9 days, while in non-adjusted treatments, *A. niger* exhibited peak activity at day 12 (Fig. 5)**.**

Both *A. niger* and *A. flavus* are well-recognized for producing a wide spectrum of extracellular enzymes, including ligninases, cellulases, amylases, oxygenases, and dehydrogenases, which enable the degradation and mineralization of complex organic substrates (Zhang et al. 2024; Sarwan et al. 2024). These enzymes are non-toxic, biodegradable, and function effectively at low concentrations (Baffi et al. 2024). Dehydrogenases are particularly important biomarkers, reflecting microbial oxidative metabolism and energy turnover, and are capable of degrading diverse pollutants, including dyes and aromatic hydrocarbons (Bhandari et al. 2021). Their activity correlates with enzymatic hydrolysis of complex carbohydrates and lignocellulosic compounds, contributing to reductions in BOD and COD.

*A. niger* additionally produces organic acids, such as citric acid, which acidify the surrounding medium and enhance both pollutant solubilization and enzyme efficiency (Kumar et al. 2025). *A. flavus*, on the other hand, synthesizes secondary metabolites that can chelate metal ions, facilitating phosphorus mobilization and removal. During organic matter biodegradation, laccase, peroxidase, and oxygenase enzymes also play key roles, further supporting pollutant breakdown (Baffi et al. 2024). Previous investigations likewise confirm the ability of *A. niger* to degrade complex organic contaminants into simpler, less toxic intermediates (Bibi et al. 2018; Gharieb et al. 2024).

Overall, the enzyme activity profiles demonstrated here confirm that both fungal species possess high metabolic adaptability and are well suited as biological agents for treating nutrient-rich aquaculture wastewater under variable environmental conditions (Li and Wang 2023).

### Pollutant removal performance and comparative fungal efficiency

Removal efficiency values were calculated using the formula:

$$removal effeciency \% = (C_{i} - C_{f} )/C_{i} x100$$

Where, Ci and Cf are the initial and final period, respectively. The data in Table 2 indicate that all *Aspergillus* treatments exhibited substantial pollutant and nutrient removal during the experimental period.

#### Total protein and dehydrogenase

Both *A. flavus* ( +) and *A. niger* removed approximately 66% of total protein within 3–9 days, indicating rapid degradation of organic nitrogen compounds. The slightly lower removal by *A. niger* ( +) (63.7%) may reflect differences in substrate affinity or metabolic adaptation. Consistent with Pobiega et al. (2024), fungal proteolytic enzymes played a critical role in reducing protein levels through enzymatic hydrolysis and biosorption, supporting biomass synthesis and detoxification.

Dehydrogenase removal was generally low (2–10%) except for *A. flavus* ( +), which exhibited moderate activity at day 6 (6.9%). Decreases in dehydrogenase over time indicate substrate depletion and reduced respiratory demand, aligning with observations by Civzele et al. (2025) in fungal wastewater systems where enzyme activity declines after major organic substrate breakdown.

#### Phosphorus, nitrogen, COD, and organic matter removal

The highest TP removal occurred with *A. niger* (93.6% between 3–6 days), suggesting strong phosphorus assimilation or precipitation. *A. flavus* ( +) removed 60.4% in the same period, while *A. niger* (+) achieved only 17.2% after 12 days, implying that nutrient enrichment may have inhibited phosphorus uptake. Similar high phosphorus removal efficiencies by filamentous fungi have been reported for *Aspergillus luchuensis* and *Trametes versicolor* (Dalecka et al. 2020), confirming the efficiency of fungal assimilation and chemical binding mechanisms. These findings parallel the present results, highlighting the strong phosphate sequestration potential of filamentous fungi.

TN removal was comparable among all treatments (54–56% between 3–6 days), reflecting early fungal nitrogen assimilation. Increases in TN at later stages may result from biomass turnover. These patterns mirror fungal nitrogen metabolism reported by Bhambri et al. (2021) for *Aspergillus* strains which achieved over 80% total nitrogen removal in industrial wastewater.

COD reduction was significant in *A. flavus* ( +) (35.9% within 3–6 days), whereas *A. niger* treatments required 12 days for maximum COD decrease, consistent with slower breakdown of complex organic matter. This aligns with findings by Serag et al. (2025) for *Aspergillus* spp. demonstrating COD reduction through fungal oxidative enzymatic pathways*.* Such activity confirms the extended metabolic capability of fungi to handle both soluble and particulate organic loads.

Organic matter removal varied sharply among treatments: *A. niger* ( +) achieved complete OM removal (100%) after 12 days, confirming strong saprotrophic and bioflocculation capabilities. Lower OM removal in other treatments (0–16%) may reflect limited enzymatic conversion or substrate saturation. This agrees with Shende and Hiwarale (2025), who noted that fungal bioflocculants enhance aggregation, sedimentation, and enzymatic breakdown of suspended organic particles.

### Plackett–Burman design (PBD)

**Fig. 6:**
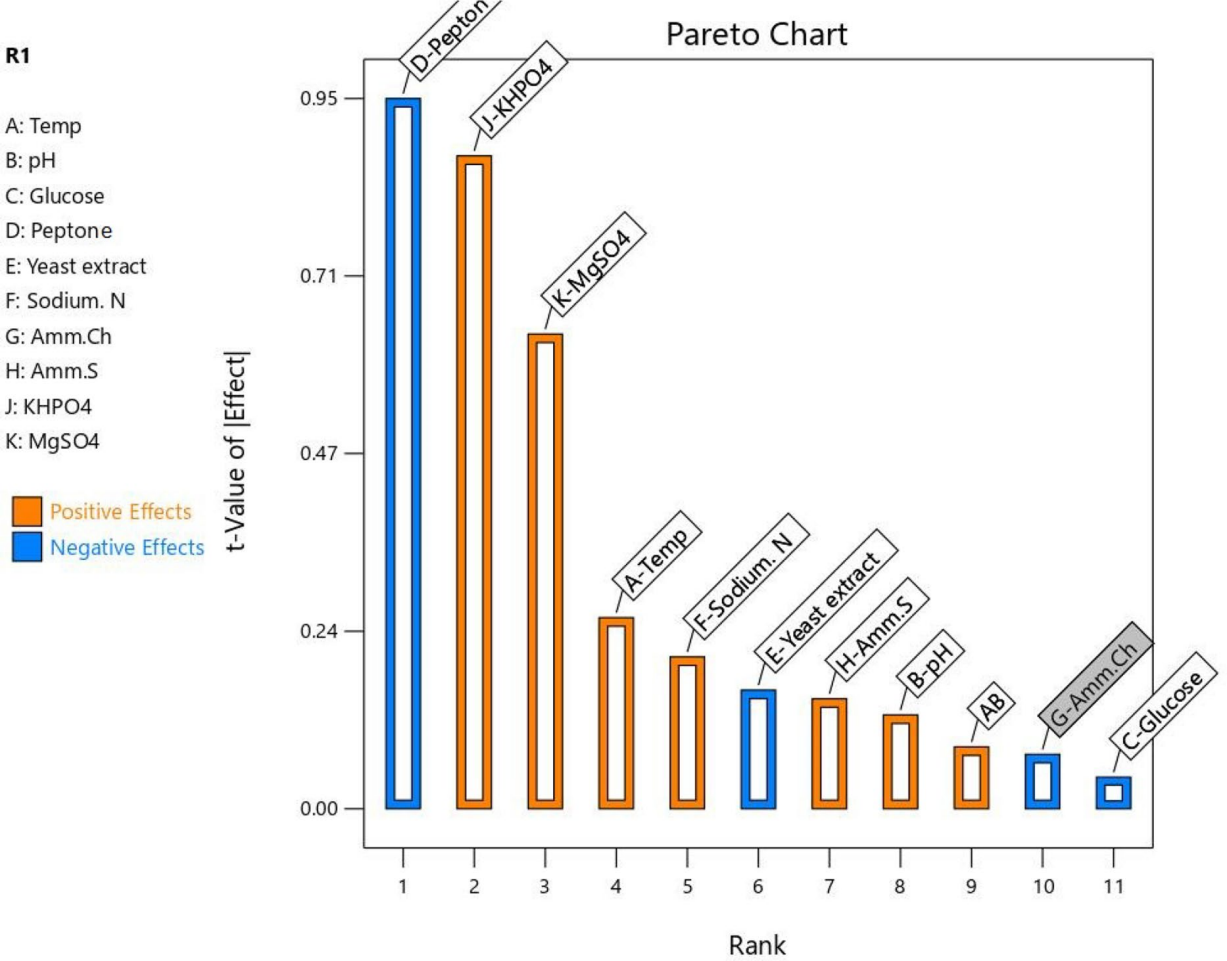
Positive and negative effects of parameters in the degradation process with Fungi

**Fig. 7:**
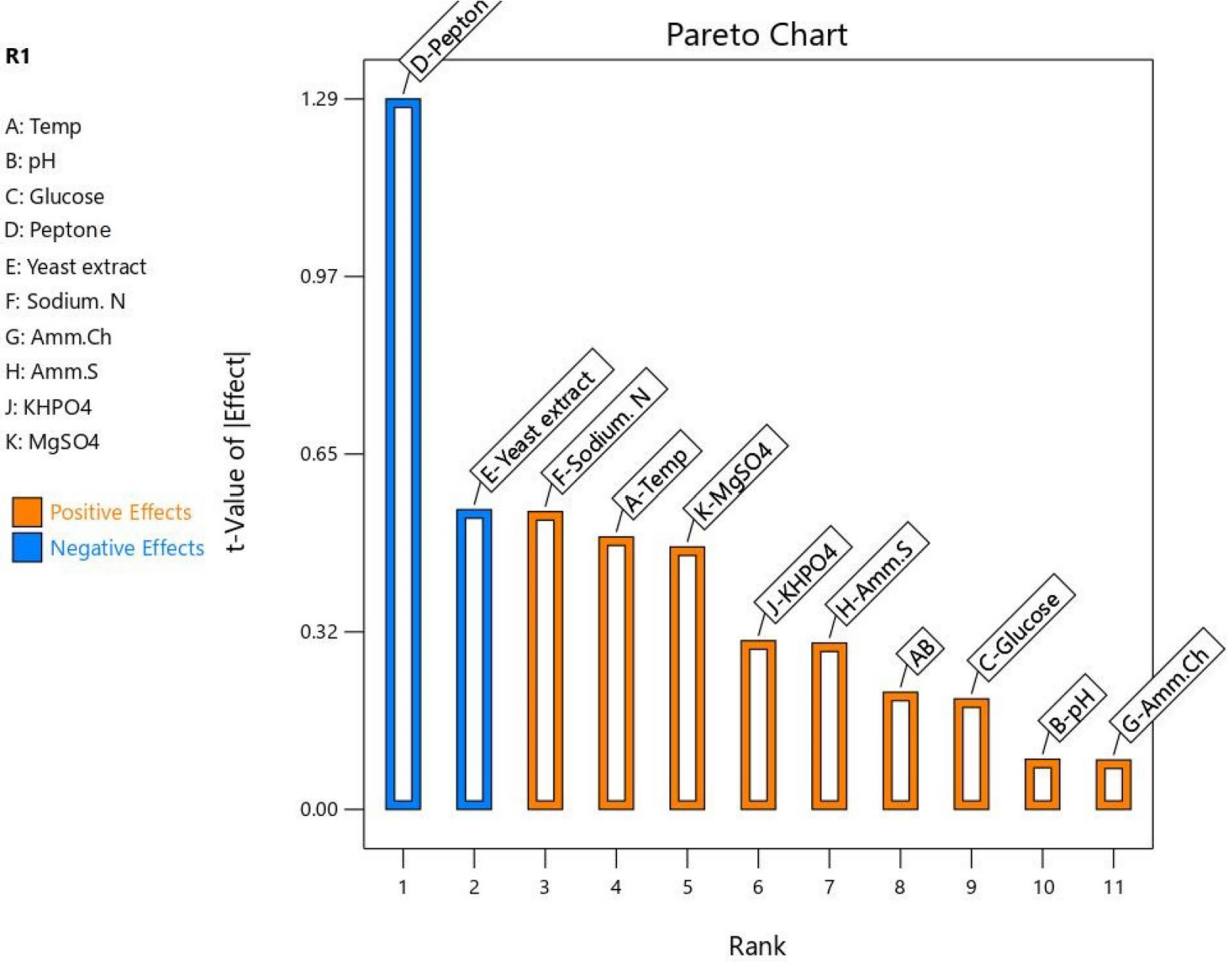
Positive and negative effects of parameters in degradation without Fungi

This study applies the Plackett–Burman design to optimize parameters influencing fungal remediation efficiency and investigates the detailed modes of action of these fungi. The placket-Burman design was applied according to data in Table 1. This is to make screening to variables (Waśko et al. 2010; Abd El-Hamid et al. 2018). The application of PB was for finding the best conditions to remove the TP from wastewaters and decreasing the eutrophication process. According to Table 1, the effect value of factor indicated the degree of its effect to the response factor. The results obtained from (PBD) screening were utilized to construct a Pareto chart (Figs. 6 and 7) for identifying significant variables.

The significant model equation in terms of coded factors for with and without conditions was as follow:

$$Y_{with} = 1.8 - 0.16*Glu\cos e - 0.83*Peptone - 0.08*Yeast + 0.73*KH_{2} PO_{4} + 0.48*MgSO_{4} + 0.05*Glu\cos e*Yeast - 0.98*Peptone*KH_{2} PO_{4}$$

$$Y_{without} = 10.69 - 0.34*Temp - 1.62*pH + 0.08*Glu\cos e - 2.11*Peptone - 0.22*Yeast + 0.55*NaNO_{3} + 0.37*NH_{4} Cl + 1.24*\left( {NH_{4} } \right)_{2} SO_{4} + 0.17*KH_{2} PO_{4} + 0.39*MgSO_{4} + 0.06*Temp*pH$$

As the non-significant factors, that hindered the design of an effective significant mathematical model representing the experimental results were excluded. The coded equation enables predictions of the response for specified levels of each factor. This coding facilitates comparison between factor coefficients to identify their relative impacts.

The model’s fit was evaluated using the coefficient of determination (R^2^). This value measures the proportion of variance in actual response data explained by the experimental factors and their interactions. The R^2^ value of 1 represents perfect fit, where the model explains all observed data variations. The R^2^ for each model was 0.99 and 1 for with and without conditions, respectively.

The analysis of the regression coefficients of the ten factors elucidated that KH_2_PO_4_, MgSO_4_, temperature, NaNO_3_, (NH_4_)_2_SO_4_ and pH have positive effects in case of fungi (*Ganoderma* sp.) (Fig. 6). The effect of KH2PO4 may due to the component of HPO4 which is a component of nucleotides having ATP and phospholipids (Fan and Makielski 1997). The KH_2_PO_4_ showed the highly positive effect. While the pH and temperature showed low to slight contribution, this result is agreed with Ahuja et al. (2004) in his study on aggregated Shipworm Bacterium. Also, potassium plays a vital role as an enzymatic activator in metabolic and physiological processes (Mehdaoui et al. 2022). It was detected that glucose and ammonium sulphate had significant effect on phosphate solubilization (Padmavathi 2015). Here ammonium sulphate showed positive effect and glucose showed negative impact. (NH_4_)_2_SO_4_ is a cost-effective source to inorganic nitrogen that is widely used in cellulose production (Jatinder et al. 2006). MgSO_4_ was selected as important nutrient based on their positive influence on enzyme formation in experiment of Francis et al. (2003). Our results showed high effect of MgSO_4_.

**Fig. 8:**
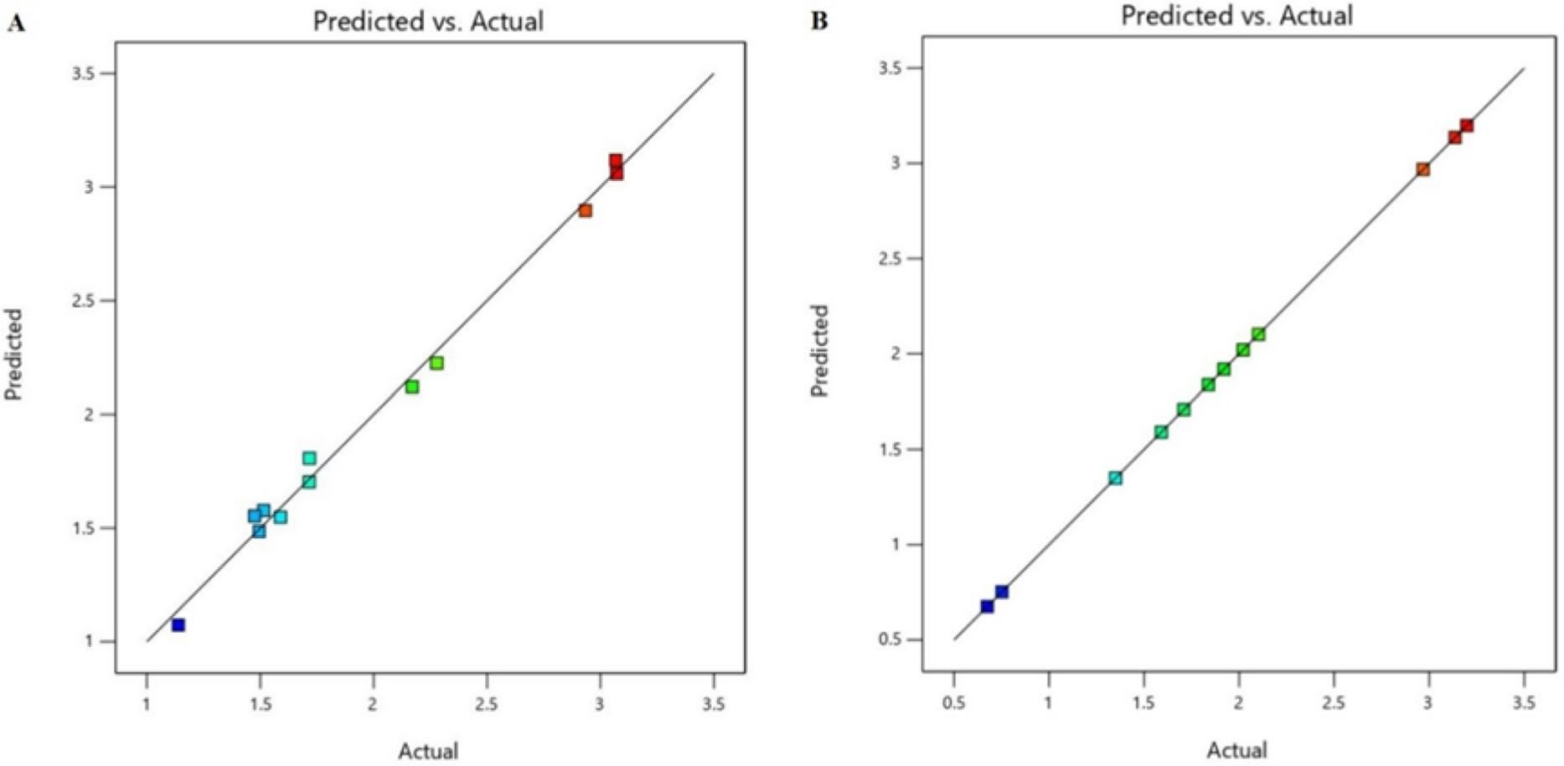
Relation between predicted and actual values; **A** with fungal conditions and **B** without fungal conditions

In absence case of fungal strains, all parameters showed positive impacts except for yeast extract and peptone (Fig. 7). Verma and Ekka (2017) reported that nitrogen sources have the positive effect on phosphate solubilization. This is agreed with the finding of results of sodium nitrates. Plackett–Burman design has also been successfully employed to optimize biosorption parameters for dye removal, as demonstrated by Abd El-Hamid et al. (2022), who achieved efficient remediation of crystal violet using *Sargassum latifolium* biomass. Here, the relation between predicted and actual values of fungal biomass in our study was as illustrated in Fig. (8).

**Fig. 9:**
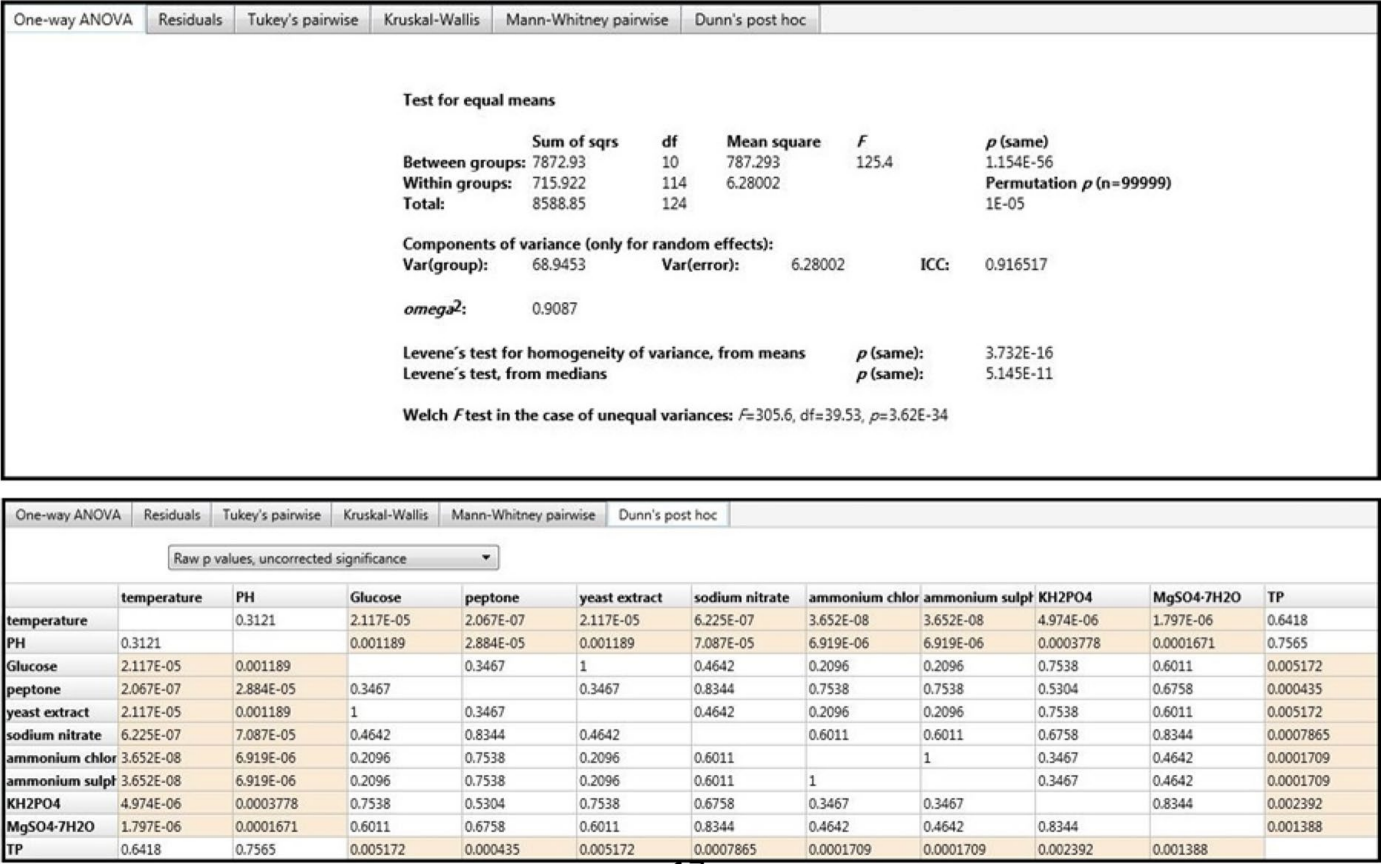
One-way ANOVA and Dunn’s post hoc analyses for factors impacted on TP removal

The One-way ANOVA analysis showed high significance difference among the tested factors (*p* < 0.001). The value of w^2^ (0.91) approved that the demonstrated model was fit in the Blacket Burman design. The individual comparisons were evaluated using the Dunn’s post hoc (Vyas et al. 2019). It showed that the most effective factors were peptone, ammonium salts, sodium nitrate *p *< 0.001); for MgSO_4_, KH_2_PO_4_, followed by Glucose and yeast extract (*p *< 0.05). Whereas, temperature and pH showed less significance (*p* > 0.05) (Fig. 9). Chen et al. (2005) found that sucrose; KH_2_PO_4_ and ZnSO_4_ were vital and effective factors for excretion of extracellular polysaccharides of *Ganoderma lucidum.* For this species of *G. lucidum*, it was obtained that glucose and yeast extract were explored to be the optimum carbon and nitrogen sources based on the single factor tests. Also it is well-known that polysaccharides and ganoderic acids are the most bioactive constituents of *Ganoderma* species (Wei et al. 2014). Though few studies in this field were obtained for *G. mbrekobenum*.

El-Sesy and Aly (2021) found that the most significant conditions for excretion of cellulosic enzymes for *A. niger* were substrate concentration, shaking, temperature, time of incubation, inoculum size and peptone concentrations. One of the limitations in *G. mbrekobenum* experiment was the used inoculum size which may impacted the removal efficiency of TP.

### Validation

The optimal model for TP removal was developed to minimize eutrophication risks, and validated through experimental verification.We refined our experimental blueprint to ensure the simulation model was shipshape. The regression models were validated based on R^2^ values. The measured TP content was very close to the predicted value (Fig. 8), proving our model’s accuracy. We conducted another experiment to test the model’s predictive prowess. The measured TP content was again remarkably close to the forecast, confirming the model’s reliability. With these results, our model stands validated, ready to guide future experiments.

Mycoremediation using *Ganoderma* species offers a promising and environmentally friendly approach to treating fish pond wastewater. It is a white rot fungus that is known for its ability to degrade certain pollutants while simultaneously producing valuable metabolites like proteins and enzymes (El-Gharabawy et al. 2016; 2022). It can produce powerful ligninolytic enzymes such as laccases, lignin peroxidases, and manganese peroxidases, which are non-specific and can break down a wide range of complex organic pollutants, including those commonly found in aquaculture wastewater (El-Gharabawy 2023; Wang et al. 2023). This process is cost-effective, environmentally friend, and effective for recalcitrant pollutants, high persistence and potential for biomass valorization (Mooralitharan et al. 2023; Tayar et al. 2025; Campos et al. 2025). In summary, *Ganoderma* holds significant potential for the mycoremediation of fish pond wastewater by breaking down organic pollutants and nutrients, and promoting flocculation. Further research and development will help realize its full potential as a sustainable and effective wastewater treatment solution for aquaculture.

*Aspergillus* spp. exhibit high biomass and enzyme yields, making it suitable for large-scale applications. In addition, they exhibit high tolerance to environmental stresses, making it suitable for diverse wastewater conditions. All these characters are very promising for wide application in industrial wastewater treatment, particularly where organic matter and recalcitrant compounds are prevalent. The fungal high enzyme production capacity allows rapid degradation and detoxification processes, particularly for aromatic and phenolic pollutants commonly found in aquaculture effluents. These enzymes can be used in immobilized form to enhance reusability and stability in bioreactors (Abd El-Hamid et al., 2015; Kaur et al. 2020; Serag et al. 2025).

The tested fungi (*A. niger*, *A. flavus*, and *Ganoderma* sp.) play crucial roles in biodegradation through diverse enzymatic mechanisms, offering a sustainable solution for fish pond wastewater treatment. Their combined use, coupled with innovations in immobilization, genetic engineering, and process design, can significantly enhance remediation efficiency. Moving forward, integrated approaches, technological advancements, and supportive policies will be essential to fully harness fungi’s potential in environmental management.

### Comparing between fungal remediation approach and other remediation techniques

The treatment of wastewaters using fungi could be improved if linked to another organism as algae as it make this more efficient (Roy et al. 2025). One of the most advantages of bioremediation is cost-effective when compared with other techniques i.e. chemical or physical processes in addition to least maintenance (Zeyaullah et al. 2009; Chakraborty et al. 2025). Tested fungi as *A. niger* and *Trichoderma sp.* can be more effective in metal ions bioleaching form marine sediments than acidophilic autotrophic and heterotrophic bacteria (Dell’Anno et al. 2022). The fungi were more efficient in decolorization of wastewater comparing to bacteria as it require preadaptation to certain pollutants (Garg and Tripathi 2011; Pakshirajan and Radhika 2013; Liu et al. 2017).

## Conclusion

This study demonstrates the strong potential of fungal biomass as an effective, low-cost, and eco-friendly agent for aquaculture wastewater treatment. The integration of mycoremediation with the Plackett–Burman statistical design provided a novel and efficient strategy for identifying the key operational factors governing pollutant removal. The results highlight the successful application of *Aspergillus niger*, *Aspergillus flavus*, and *Ganoderma mbrekobenum* in fungal-mediated remediation of nutrient-rich aquaculture effluents.

All fungal treatments contributed to significant reductions in total nitrogen (TN), total phosphorus (TP), chemical oxygen demand (COD), and oxidizable organic matter (OOM) within 9–12 days of incubation. Concurrent increases in dehydrogenase and total protein levels during this period indicate enhanced fungal metabolic activity and biodegradative capacity. These findings confirm that bioremediation using native *A. niger* and *A. flavus* can effectively improve the ecological quality of polluted water bodies by reducing key physicochemical and nutrient parameters in a relatively short treatment timeframe.

The Plackett–Burman design identified KH₂PO₄ and MgSO₄ as major enhancers of phosphorus removal in the presence of fungal biomass, while peptone and pH exerted greater influence in non-fungal systems. The regression model demonstrated high predictive reliability (R^2^ > 0.99), confirming the robustness of the optimization approach. Comparative analysis revealed that *A. niger* exhibited superior removal efficiencies for TP and TN, while *A. flavus* showed stronger enzymatic performance in degrading complex organic pollutants (COD). In the Plackett–Burman experiment, *G. mbrekobenum* demonstrated TP removal influenced by ammonium salts, peptone, and glucose concentrations, indicating that fungal species differ in remediation capacity depending on pollutant type and environmental conditions.

Overall, the findings confirm that fungal bioremediation, supported by statistical optimization, represents a cost-effective, sustainable, and environmentally compatible strategy for treating aquaculture wastewater. This approach has the potential to reduce the ecological footprint of aquaculture activities and support the protection and restoration of vulnerable aquatic ecosystems. Future work will focus on pilot-scale system validation under field conditions, detailed profiling of enzymatic pathways involved in nutrient and organic matter degradation, and integrating fungal treatment with complementary biological and physicochemical processes to enhance nutrient removal efficiency and operational stability.

## Data Availability

All data generated or analysed during this study are included in this published article.
